# Prevalence of Domestic Violence in Hong Kong Chinese Women Presenting with Urinary Symptoms

**DOI:** 10.1371/journal.pone.0159367

**Published:** 2016-07-18

**Authors:** Wai Sze Paulin Ma, Ting Chung Pun

**Affiliations:** Department of Obstetrics & Gynecology, Queen Mary Hospital, Hong Kong SAR, China; Medical University of Vienna, AUSTRIA

## Abstract

**Objectives:**

To determine the prevalence of domestic violence and its risk factors in women presenting with urinary symptoms.

**Methods:**

The study was carried out in the urogynecology clinic and general gynecology clinic, Department of Obstetrics and Gynecology, Queen Mary Hospital, Hong Kong from 1^st^ May 2013 till 31^st^ October 2014. Two hundred and twenty-five women presenting to the urogynecology clinic with urinary symptoms were categorized according to their symptoms and were asked to complete the Modified Abuse Assessment Screen. Demographic data of the subjects and their partners were collected. Mann-Whitney U test were used for analysis of continuous variables, while Chi-square test and Fisher Exact test were used for analysis of categorical variables between the abused and non-abused group. Prevalence of domestic violence were calculated and compared.

**Results:**

The prevalence of domestic violence among this group of patients (7.6%) was found to be lower when compared with other studies. Verbal abuse was the commonest form of violence in our locality. The median age of the abused group and the non-abused group were both 56 years old, with the age ranging from 40 to 64 and 29 to 70 years old respectively. The prevalence of domestic violence among patients with overactive bladder syndrome, stress urinary incontinence and mixed urinary incontinence were 19.5%, 4.2% and 5.5% respectively (Fisher Exact test for whole group, *P*<0.05).

**Conclusion:**

The prevalence and nature of abuse in our locality was different from the quoted figures worldwide. Patients with overactive bladder syndrome were more likely to be victims of abuse than patients with other urinary symptoms. The difference in the prevalence of domestic violence among patients with different urinary symptoms could be related to their underlying pathophysiology. When encountering patients with overactive bladder syndrome, clinicians should consider this high incidence of domestic violence and provide prompt referral whenever necessary.

## Introduction

Domestic violence is a common and important issue worldwide. Its reported prevalence in USA varied from 6.3% in a health care survey to 55% in women attending primary care clinic. [1–5] In the World Health Organization (WHO) multi-country study on women’s health and domestic violence against women, data from over 24000 women in 10 countries were collected. [6] Domestic violence is classified into physical, sexual and emotional (psychological). Act of physical violence includes slapping, hitting, kicking and beating, while sexual violence includes forced sexual intercourse or any form of sexual coercion. Insults, belittling, constant humiliation, intimidation and threat of harm are considered as action of emotional (psychological) abuse. [7] The severity of physical violence was also measured in the report. Being slapped, pushed and shoved were considered as moderate physical violence, while being hit by a fist, kicked, dragged, threatened by a weapon, or attacked by a weapon were defined as severe physical violence. [6] It was found that 13–61% of women suffered from physical abuse. Among this group of women, 4–49% of them experienced severe physical abuse from their partners. Sexual abuse and emotional abuse were also common. The incidences were reported to be up to 59% and 75% respectively. [6] Local studies performed in Hong Kong showed that the prevalence of domestic violence in Hong Kong ranged from 10.4% to 15.7%. [8] “Battering syndrome” is suggested to occur in victims of domestic violence, in which physical assault is followed by increase in general medical and emotional problems. [9] In a cross-sectional study, victims of abuse showed a higher rate of non-specific lower abdominal pain and bowel irritation when compared to those who did not suffer from abuse. [10] Studies have also demonstrated a relationship between urological symptoms and abuse. However, the targeted group of the studies were mainly children and adolescence, with only a few focused on women who suffered from domestic violence. Among the child victims, sexual abuse was found to be associated with lower urinary tract symptoms. In one study, dysuria was reported by 47.7% of victims of sexual abuse compared to 24.7% of those who did not report experience of sexual abuse. (*P*<0.003) [11] Similar findings were also reported by another study, with urinary incontinence and urgency as the reported symptoms. [12] Latest study has extended to the relationship between bullying and lower urinary tract symptoms in children. More children in the bullying group reported urinary symptoms. [13] The higher anxiety level in the abuse victims was suggested to play a role in the development of urinary symptoms. [13]

High anxiety level within the victim of abuse could be seen in their adult counterpart. Women who reported current violence were 4.81 times more likely to report psychological distress. [14] Major depressive disorders was more common in women who have experience of abuse (29.6% in violence victims versus 16.5% in control group, *P*<0.002) [15] If the underlying pathophysiology of urinary symptoms were similar in both children and adults, it would be expected that women suffering from domestic violence would also be more prone to development of urinary symptoms. In one study which involved 243 women who attended the obstetrics and gynecology clinic, the prevalence of physical or sexual abuse in patients with overactive bladder is higher than those presenting with stress urinary incontinence, with a percentage of 30.6% and 17.8% respectively (*P*<0.05). [16] Another study showed the positive association of urinary symptoms with sexual, emotional or physical abuse; with an odd ratio of 2.09, 2.28 and 2.36 respectively for the symptoms of urinary urgency. The odd ratio for urinary frequency in those experienced with abuse was found to be ranging from 1.56 to 1.97 (*P*<0.05). [17] It was also suggested that victims of abuse suffered from fear and anxiety which may manifest as physical symptoms, especially sexual or urinary complaints, even years after the abuse has occurred. [17]

Another questionnaire survey by a group in the United Kingdom tried to study the specific gynecological symptoms or referral reasons in patients who reported on emotional abuse. Termination of pregnancy, abnormal cervical smear, worry about cancer and stress incontinence were the more common reasons for referral in women who had emotional abuse compared to those who had no such experience. Seven percent (14 out of 198) abused women complained of stress incontinence whereas only 3% (19 out of 627) of those who had no experience of abuse complained of the same symptoms. [18] Urinary tract infection was another urinary symptoms being evaluated, with an age-adjusted relative risk of 1.79 (95% CI 1.36–2.36) in patients with history of abuse. [19]

The presentation of domestic violence is often cultural specific. From previous studies in our locality, it was found that the majority of abuse was in the psychological and emotional aspects with verbal intimidation. [7,20] The most effective way in identifying domestic violence is to routinely ask all female patients about it, since most women do not volunteer the history of abuse due to fears and concerns about the negative consequences of reporting. [3] Time constraints and lack of prioritization generally limit this line of questioning in clinic. Presence of symptoms related to domestic violence might raise doctors’ suspicion and lead to an initial enquiry.

We hypothesized that the psychological impact of domestic violence would raise the susceptibility of urinary symptoms in the victims. Prior researches have focused on child violence exposure and for those whose subjects of research were adults, they were limited by the lack of standardization in classification of the urinary symptoms.

This study addresses this gap by studying the prevalence of domestic violence in women presenting with urinary symptoms which were classified by the standardized categorization according to the International Continence Society. [21] The associated risk factors of domestic violence in this group of patients will also be studied.

## Materials and Methods

Between 1^st^ May 2013 and 31^st^ October 2014, all Chinese women, who were able to read Chinese, aged between 18 years old and 70 years old who presented with urinary symptoms to the Urogynecology Clinic and General Gynecology Clinic, Department of Obstetrics and Gynecology, Queen Mary Hospital, Hong Kong, were invited to join the study. Patients who wished to withdraw from study or had mental impairment were excluded. Written consent was obtained from the women before filling in the questionnaire and data collection. This study was approved by the local Institutional Review Board (Hong Kong West Cluster, Queen Mary Hospital; IRB Ref. No UW 13–301).

The medical records of the patients who were recruited in the study were reviewed. They were divided into three groups: Overactive bladder (OAB) group, Stress Urinary Incontinence (SUI) group and Mixed Urinary Incontinence (MUI) group, according to the doctor’s diagnosis which were based on the classifications of symptoms as suggested by the International Continence Society. [21]

As described in detail previously in our study on domestic violence in patients attending colposcopy clinic [22], subjects were interviewed by a designated research nurse in a private setting in the absence of male partners or husbands. The five-item Modified Abuse Assessment Screen was used as tool for screening for domestic violence. This validated questionnaire has been used in our previous series of studies and was found to be a sensitive and reliable questionnaire in identifying domestic violence. [7,20] Women who answered “yes” to question 1 “Have you ever been emotionally or physically abused by your partner or someone important to you” were considered victims of domestic violence. Question 2 ‘Within the past year, have you been hit, slapped, kicked or otherwise physically hurt by someone?’ and question 3 ‘Within the past year, has anyone forced you to have sexual activity?’ were used to assess the nature of the abuse experienced by the victims.

Demographic data including age, marital status, duration of present marriage, education level of women and their partners, occupation of women and their partners, parity, religion and the total family income were recorded by the research nurse. In our study, parity is defined as the number of live-born children and stillbirths a woman has delivered at more than 20 weeks. Occupation of women and their partners were divided into manual, clerical and professional. Manual work referred to jobs that required mainly physical work while clerical work referred to office work and administrative support duties. Professional was considered as occupation in which standard of education and training were needed.

Statistical analysis was performed using the SPSS/PC software package version 20.0. Descriptive statistics were used to summarized the characteristics of patients screening positive and negative for domestic violence and were expressed as numerical values and percentages. Study variables were tabulated by experience of abuse (the abuse group) and those without experience of abuse (the non-abused group). Mann-Whitney U test was used in comparing the continuous variables, in this study, the age and years of marriage between the two groups. Chi-square test was used to quantify any differences in the categorical demographic data between the abused and non-abused group. Fisher’s exact test was used to determine the relationship between exposure to domestic violence with different categories of urinary symptoms: stress urinary incontinence; overactive bladder syndrome and mixed urinary incontinence. Statistical significance was considered as *P*≤0.05.

## Results

**Fig 1:**
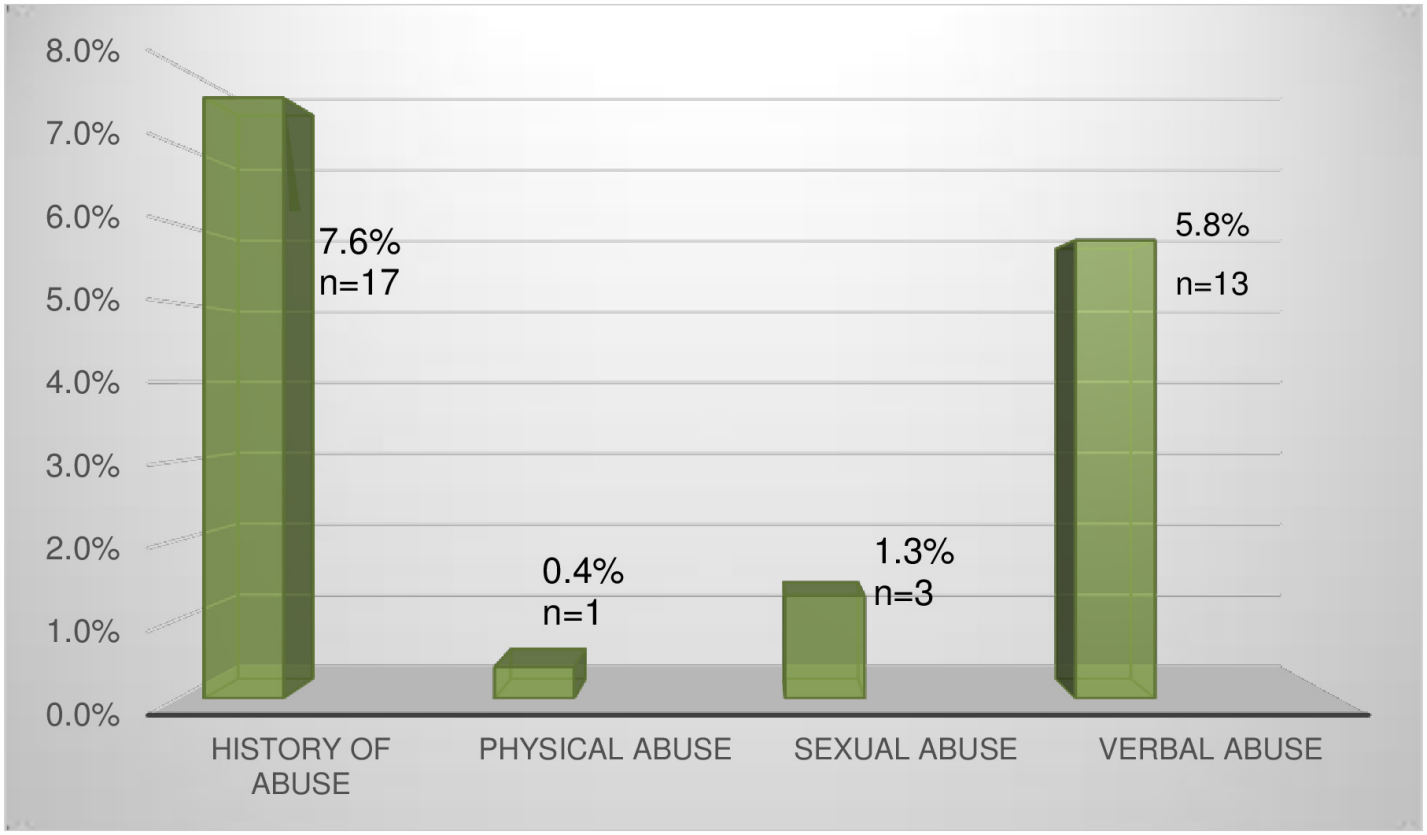
Prevalence and Distribution of Domestic Violence.

During the study period, two hundred and thirty-four women attended the clinic and were eligible for study. Nine of them (3.8%) refused to participate in the study. Seven refused as they did not want to wait for the interview by the research nurse. Two refused without giving any reason. Two hundred and twenty-five women were interviewed. The response rate was 96.2%. Seventeen of them (7.6%) reported history of domestic violence. Thirteen (5.8%) suffered from verbal abuse while the incidence of physical abuse and sexual abuse were 0.4% and 1.3% respectively. The prevalence and distribution of domestic violence are shown in Fig 1.

Demographic data of women with and without history of abuse were compared (Table 1). The median age of the abused group and non-abused group were identical, at 56 years. When further subdivided the participants according to their age group, the incidences of domestic violence were similar, ranging from 6.5 to 9.4%. Among all the demographic data, marital status was found to be a possible risk factor for domestic violence. The incidence of abuse is higher among those who are divorced (20%), twice more common than in those who were married, although it did not reach statistical significance (*P* = 0.07).

**Table 1:** Demographic factors for women with or without history of domestic violence.

	Abused (%) (n = 17)	Non-abused (%) (n = 208)	*P*-value in comparing abused and non-abused group	Prevalence of abuse (%)
Age (years)	56.00	56.00	0.96 *	—
Median [range in years]	[40–64]	[29–70]		
Age group (years)			0.94	
<45	2 (11.8)	24 (11.5)		7.7
45–49	2 (11.8)	29 (13.9)		6.5
50–54	2 (11.8)	39 (18.8)		4.9
55–59	5 (29.4)	48 (23.1)		9.4
≥60	6 (35.2)	68 (32.7)		8.1
Marital Status			0.07	
Married	13 (76.5)	161 (77.4)		7.5
Single	0	13 (6.3)		0.0
Widowed	0	18 (8.6)		0.0
Divorced	4 (23.5)	16 (7.7)		20.0
Duration of present marriage (years)	32.5	29.5	0.23	—
Median [range in years]	[3–44]	[4–55]		
Parity			0.28	
0	4 (23.5)	28 (13.5)		12.5
≥1	13 (76.5)	180 (86.5)		5.9
Educational Level			0.43	
Below primary	3 (17.6)	18 (8.7)		14.3
Primary	4 (23.5)	43 (20.7)		8.5
Secondary	7 (41.1)	117 (56.2)		5.7
Tertiary	3 (17.6)	30 (14.4)		9.1
Employment			0.45	
Yes	7 (41.2)	109 (52.4)		6.0
No	10 (58.8)	99 (47.6)		9.2
Employment			0.59	
Manual	3 (17.7)	55 (26.4)		5.2
Clerical/ Professional	4 (23.5)	54 (26.0)		6.9
Unemployed	1 (5.9)	5 (2.4)		16.7
Retired/ Housewife	9 (52.9)	94 (45.1)		8.7
Religion			1.00	
Yes	6 (35.3)	69 (33.2)		8.0
No	11 (64.7)	139 (66.8)		7.3

Characteristics of the partners were also analyzed. No significant difference in prevalence of domestic violence was found among partners with different education level, employment status, nature of occupation or total family income. The results are shown in Tables 2, 3 and 4.

**Table 2:** Educational level of partners.

	Abused (%) (n = 15)	Non-abused (%) (n = 171)	*P*-value when comparing abused to non-abused group	Prevalence of abuse (%)
Educational Level (n = 186)			0.52	
Below primary	2 (13.3)	8 (4.8)		20.0
Primary	2 (13.3)	31 (18.1)		6.1
Secondary	8 (53.3)	102 (59.6)		7.3
Tertiary	3 (20.0)	30 (17.5)		9.1

**Table 3:** Occupational status of partners.

	Abused (%) (n = 12)	Non-abused (%) (n = 167)	*P*-value when comparing abused to non-abused group	Prevalence of abuse (%)
Employment (n = 179)			0.76	
Yes	9 (75.0)	115 (68.9)		7.3
No	3 (25.0)	52 (31.1)		5.5
Employment			1.00	
Manual	5 (41.7)	62 (32.1)		7.5
Clerical/ Professional	4 (33.3)	53 (31.7)		7.0
Unemployed	0	7 (4.2)		0
Retired	3 (25.0)	45 (27.0)		6.3

**Table 4:** Total family income.

	Abused (%) (n = 16)	Non-abused (%) (n = 207)	*P*-value when comparing abused to non-abused group	Prevalence of abuse (%)
Income (HK$)			0.44	
<5000	1 (6.3)	19 (9.2)		5.0
5001–10000	4 (25.0)	26 (12.6)		13.3
10001–20000	5 (31.3)	43 (20.8)		10.4
20001–30000	2 (12.5)	45 (21.7)		4.3
>30000	4 (25.0)	74 (35.7)		5.1

The association between the nature of urinary symptoms and domestic violence is shown in Table 5. Prevalence of abuse in patients categorized as stress urinary incontinence group, mixed urinary incontinence group and overactive bladder group were found to be 4.2%, 5.5% and 19.5% respectively. The incidence was significantly higher in patients presenting with overactive bladder symptoms (Fisher Exact test, *P*<0.05).

**Table 5:** Urinary symptoms in abused and non-abused groups.

Urinary symptoms	Abused (%) (n = 17)	Non-abused (%) (n = 208)	*P*-value when comparing abused to non-abused group	Prevalence of abuse (%)
Urinary symptoms			0.02	
Stress urinary incontinence	1 (5.9)	23 (11.1)		4.2
Mixed urinary incontinence	9 (53.0)	156 (75.0)		5.5
Overactive bladder syndrome	7 (41.2)	29 (13.9)		19.4

## Discussion

The reported prevalence of domestic violence ranges from 6.3% to 55% in USA. [1–5] Local studies have reported the incidence of domestic violence in Hong Kong to range from 10.4% to 15.7%. [7,20] Identification of domestic violence is difficult as women may not volunteer the history of abuse in view of fears and concerns about the negative consequences of reporting. [3] In our study, urinary symptoms were examined as suggestive of possible abuse as previous studies showed positive correlation between domestic violence and urinary symptoms. [16–17] It was proposed that the manifestation of sexual or urinary complaints, even years after the abuse, could result from the anxiety experienced by the abused patients. [17] In our study, only 7.6% of women attending urogynecology clinic complaining of urinary symptoms who agreed to participate in the study were victims of domestic violence, which is lower than the figures quoted from previous studies. The nature of abuse was also found to be different from other localities. Sexual violence was reported to be significantly associated with urinary incontinence in Malawi and Rwanda. [23] However, verbal abuse was found to be a more common form of abuse in our locality. Chinese culture was much influenced by Confucianism and Buddhism. With these beliefs, being greedy; telling lies and hatred are all considered as deviations from ideal. Therefore, anyone who expresses these ideas in speech could be considered as committing a violent act. [22] Verbal abuse may contribute to adverse psychological impacts on patients even though it did not cause any physical damage and resulted in high anxiety level in victims. [18] In some literatures, high anxiety level was found to be one of the contributing factors to the presentation of urinary symptoms. [24–25] Our study also demonstrated the relationship of domestic violence with different categories of urinary symptoms. The significant finding from our study is that there was higher percentage of domestic violence among patients presenting with overactive bladder syndrome when comparing to those with stress urinary incontinence and mixed urinary incontinence (19.4% versus 4.2%/ 5.5%, Fisher exact test for whole group, *P*<0.05).

Since the 1970s, studies have sought relationships between psychological stress and urinary symptoms. Positive correlation was found between bladder dysfunction and psychological trauma. [26–27] However, the exact mechanism of psychological stress as the causative factor for overactive bladder symptoms was not elaborated. Recently, autonomic nervous system dysfunction was suggested as a causative factor of overactive bladder symptoms. Heart rate variability, cardiovascular tests and sympathetic skin responses were used as indicators of the function of the autonomic nervous system. It was found that patients with overactive bladder symptoms experienced a decrease in heart rate variability; an increase in sympathetic tone; or an impaired sympathetic skin response when compared to the control group. [28–30] Researchers thus postulated that autonomic nervous system dysfunction should be considered as a cause for overactive bladder syndrome.

This emergent idea could shine light on the correlation between domestic violence and overactive bladder syndrome. Patients who experience violence or abuse during childhood have been found to have a higher rate of chronic health problems. [31] A recent study further illustrated the higher prevalence of childhood sexual trauma in patients with OAB. Anxiety, physical symptom burden and psychological stress were reported to be significantly higher within the OAB group. [32] Prolonged stress is believed to be one of the causative factors of the urinary symptoms in patients with OAB. The hypothalamic-pituitary axis (HPA) regulates the sympathetic division of the autonomic nervous system at times of stress. In order to react with a “fight or flight” response, the cortisol hormone would increase. This could be helpful in short term, however, when there is prolonged elevation of cortisol level within the body, many of the body systems would be adversely affected. Women with a history of abuse have been shown to have a higher level of evening cortisol and morning and evening dehydroepiandrosterone. [33] Similar findings were also noted in patients with history of abuse during childhood. [34] HPA dysregulation appears to be a common pathway in the natural history of patients experiencing domestic violence and those complaining of overactive bladder syndrome. We postulated that patients with domestic violence experienced prolonged stress which may in turn lead to dysregulation of the HPA. This dysregulation could cause urinary symptoms as experienced in patients with overactive bladder syndrome.

In addition to comparing patients with stress urinary incontinence and those with overactive bladder syndrome, a new group of mixed urinary incontinence was also included in our analysis, unlike in previous studies. Its incidence was similar to those presented with stress urinary incontinence.

Differences in the underlying pathophysiology could be the reason for this finding. Weakening of pelvic floor is the cause for stress urinary incontinence while involuntary contraction of the bladder is the cause for overactive bladder syndrome. Although being the subject of investigation for many years, the pathophysiology of mixed urinary incontinence remains unclear. Fluid within urethra or distension of proximal urethra can lead to detrusor hyperactivity in animals and humans. [35–36] This is further supported by studies which showed improvement in urgency symptoms in patients with mixed urinary incontinence after treating the stress urinary incontinence with suburethral tape. [37] Taken all these into account, it is believed that although patients with mixed urinary incontinence and those with overactive bladder syndrome shared some common presenting symptoms, the underlying pathophysiology was different. Instead of being related to the HPA dysregulation, the urgency and urinary frequency in patients with mixed urinary incontinence could result from the pre-existing stress urinary incontinence. Our study provides new evidence to support this possibility. Instead of studying the mechanics of urinary incontinence and detrusor hyperactivity, we studied the association between domestic violence and different types of urinary symptoms. From the difference in incidence of abuse among these groups of patients, it is believed that there is more commonality in pathophysiology between mixed urinary incontinence and stress urinary incontinence.

Divorce and lower educational level of patients and their partners are well established risk factors of abuse [38–39]. A similar trend was demonstrated in our study, although not reaching statistical significance.

There are limitations in our study. Firstly, there were missing data on the women’s partners/ husbands; this may interfere with the data analysis. The reported prevalence should be interpreted with caution because of the possibility of underreporting. Shame, stigma, tolerance and preservation of family harmony are common shared attitude among different ethnic groups within Asia. [40] Nevertheless, pronounced interethnic differences in attitude towards abuse was found among different Asian subgroups. Chinese, Korean and Cambodian were found to be more reluctant in reporting of the abuse when compared to their Vietnamese and South Asian counterparts. [41] which could be due to the cultural norms against disclosing family issues and seeking help outside the family. [40]

Despite the limitations mentioned, to the best of our knowledge, this is the first study reporting the prevalence of domestic violence in patients with urinary symptoms attending urogynecology clinic in the Chinese population. Adding to the existing knowledge in comparing those presented with stress urinary incontinence and overactive bladder syndrome, patients with mixed urinary incontinence were also included and studied. When comparing the prevalence detected in our study with other studies carried out at family or general clinics, one may argue that screening is more cost effective in primary care setting. However, we believed that taking every chance in identifying the at-risk group is worthwhile not only due to its possible devastating consequences, but also because victims of domestic violence may enter a viscous cycle if not dealt with properly. On one hand, domestic violence could lead to presentation of different somatic complaints; patients with chronic disabilities, in particular maternal disabilities, could trigger violence due to the inability of the women to perform household chores and satisfied husband’s sexual demands. [42] Addressing this group of patients may help in breaking the viscous cycle. Furthermore, in our locality, more than half of the patients with urinary symptoms seek medical advice directly from private specialists or public hospitals, [43] therefore the at-risk group of domestic violence could be missed if screening is only applied to primary care clinics. Instead tertiary centers could be an appropriate place to apply screening for domestic violence. Taking into consideration the risk factors identified in this study and the limited resources in usual clinical practice, the detection rate in screening could be increased if overactive bladder symptoms are specially targeted, along with divorce and with lower educational levels. Addressing the possible psychological components for the somatic symptoms in this group of patients could also improve the rapport and success in management.

## Supporting Information

S1 File Modified Abuse Assessment Screen. (DOCX)
