# Supplementary material for: Prevalence of Domestic Violence in Hong Kong Chinese Women Presenting with Urinary Symptoms
Source: PLoS One. 2016 Jul 18;11(7):e0159367. doi: 10.1371/journal.pone.0159367 (PMC4948835; doi:10.1371/journal.pone.0159367)
Supplement: S1 File — (DOCX) [file pone.0159367.s001.docx]

**家庭暴力問卷調查**

| 1. 妳的配偶或妳認識的人曾否對妳作出身體上或精神上的傷害?  (1) 有  (0) 否 |
| --- |
| 2. 在過去一年裏, 妳有否被打, 掌摑, 踢, 或受到其他身體上的傷害?  (1) 有  (0) 否  如有, 對妳作出傷害的人是: (1) 丈夫  (2) 前夫  (3) 男朋友  (4) 陌生人  (5) 其他 (請註明) ______________________  被傷害的次數 ( ) |
| 3. 在過去一年裏, 曾否有人強迫妳發生性行為?  (1) 有  (0) 否  如有, 對妳作出傷害的人是: (1) 丈夫  (2) 前夫  (3) 男朋友  (4) 陌生人  (5) 其他 (請註明) ______________________  被傷害的次數 ( ) |
| 4. 妳是否害怕以上對妳作出傷害的人?  (1) 是  (0) 否 |
| 5.如妳於問題(1),(2),(3)的答案是「有」,妳是否希望我們把妳曾被虐待的資料告訴以下人士?  (a) 妳的婦科醫生 (1) 是 (0) 否  (b) 醫務社工 (1) 是 (0) 否 |

**Modified Abuse Assessment Screen**

| 1. Have you ever been emotionally or physically abused by your partner or someone important to you?  (1) Yes  (0) No |
| --- |
| 2. Within the past year, have you been hit, slapped, kicked or otherwise physically hurt by someone?  (1) Yes  (0) No  If yes, by whom? (1) Husband  (2) Ex-husband  (3) Boyfriend  (4) Strangers  (5) Others, please specify ________  Number of times ( ) |
| 3. Within the past year, has anyone forced you to have sexual activities?  (1) Yes  (0) No  If yes, by whom? (1) Husband  (2) Ex-husband  (3) Boyfriend  (4) Strangers  (5) Others, please specify ________  Number of abuse ( ) |
| 4. Are you afraid of your partner or anyone you listed above?  (1) Yes  (0) No |
| 5. Do you want us to reveal this information to: (for those answered yes to question 1,2 or 3)  (a) Your gynecologist (1) yes (0) no  (b) Medical Social Worker (1) yes (0) no |
